# High unprocessed beef intake (>100 g/d) linked to elevated triglyceride levels: evidence from cross-sectional and Mendelian randomization in Chinese and American adult populations

**DOI:** 10.3389/fnut.2026.1790394

**Published:** 2026-05-14

**Authors:** Ziyu Yi, Shuying Ding, Xiangyang Liu, Shuai Liu, Zhenyan Fu

**Affiliations:** First Affiliated Hospital of Xinjiang Medical University, Urumqi, Xinjiang, China

**Keywords:** dietary guidelines, dose–response, gene-diet interaction, Mendelian randomization analysis, triglyceride, unprocessed beef intake

## Abstract

**Background:**

Red meat, particularly beef, is rich in saturated fatty acids, which are major precursors for triglyceride (TG) synthesis. However, the association between red meat consumption and cardiometabolic risk remains controversial, and high-quality evidence is still limited. Therefore, this study aims to systematically evaluate the relationship between beef intake and TG levels in adult populations, as well as to explore potential causal links between them.

**Method:**

This study integrated data from the National Health and Nutrition Examination Survey (NHANES, 2007–2018) and the “Comprehensive Health Check-up Project” (2024) in Urumqi, China, including 2,134 and 1,974 adults, respectively, for cross-sectional analysis. A multivariable stepwise regression model was used to assess the association between beef intake (analyzed as both continuous and quartile variables) and TG levels. RCS analysis was performed to explore nonlinear relationships, and subgroup analyses were conducted to identify population-specific associations. Additionally, two-sample Mendelian randomization analysis was applied to infer a potential causal relationship from a genetic perspective.

**Result:**

Cross-sectional analyses showed that after full adjustment for confounding factors, each 100 g/d increment in beef intake was associated with an average increase in TG levels of 0.11 mmol/L in U.S. adults and 0.12 mmol/L in Chinese adults. RCS analysis revealed that the risk of hypertriglyceridemia tended to rise when beef intake exceeded approximately 100 g/d. Mendelian randomization analysis further supported a causal link, demonstrating that genetically predicted higher beef intake was associated with an approximately 2.1-fold increased risk of hypertriglyceridemia (pooled OR = 2.13, 95% CI: 1.63–2.79).

**Conclusion:**

Higher beef intake (particularly > 100 g/d) is significantly associated with elevated TG levels, and this association may reflect a genetically mediated causal relationship.

## 1 Introduction

Triglyceride (TG), composed of glycerol and three fatty acids, are the body’s primary form of stored energy. Under normal conditions, they are stored in adipose tissue and broken down during fasting or exercise to release energy for use throughout the body (1). Beyond energy supply, TG contribute to organ protection, temperature regulation, transport of fat-soluble substances, and maintenance of cell structure (2). However, when fasting TG levels remain above 1.7 mmol/L, the risk of atherosclerosis and coronary heart disease increase (3), and levels exceeding 5.6 mmol/L can raise the likelihood of acute pancreatitis (4). Chronically elevated TG are often seen in metabolic syndrome (5), obesity (6), and diabetes (7). Conversely, persistently low TG levels are also clinically significant, as they may reflect malnutrition, chronic illnesses such as cancer or hyperthyroidism, or severe liver dysfunction (8). Such conditions can impair the transport of fat-soluble nutrients and compromise cell membrane integrity. Current management of high TG emphasizes lifestyle changes over medication. Dietary recommendations include reducing saturated and trans fats and refined sugars, while increasing fiber and omega-3 fatty acid (9). When pharmacotherapy is needed, fibrates, prescription omega-3 formulations, and niacin are commonly used to lower TG, reduce pancreatitis risk, and improve cardiovascular outcome (10). Although TG have historically been considered less atherogenic than low-density lipoprotein cholesterol (LDL-C), significant cardiovascular risk remains even after achieving LDL-C targets with statins (11). Growing evidence suggests that this residual risk is closely tied to remnant cholesterol, a product of TG-rich lipoprotein metabolism. Remnant cholesterol can accumulate in the arterial wall, promoting foam cell formation, inflammation, and thrombosis, thereby accelerating atherosclerosis. Large epidemiological studies confirm that elevated remnant cholesterol independently increases cardiovascular risk (HR = 1.32), regardless of LDL-C levels (12). Thus, modern lipid management has shifted from focusing solely on LDL-C to a more integrated approach that includes TG and remnant cholesterol, reflecting their important role in cardiovascular health.

Red meat, generally referring to the muscle tissue of mammals that appears red before cooking, is an important source of high-quality protein, iron and B vitamins, but its health impact has long been controversial. In 2015, the World Health Organization’s International Agency for Research on Cancer (IARC) classified red meat as “probably carcinogenic to humans” (13). Multiple studies have since reported positive associations between red meat intake and the risk of several cancers, including colorectal, gastric, and breast cancer (14). More recent evidence also links red meat consumption to a higher risk of cardiovascular disease and diabetes (15). A notable shift in dietary guidance emerged with the release of the *Dietary Guidelines for Americans, 2025–2030* (16). Unlike previous editions that advised limiting red meat and choosing low-fat options, the latest guidelines place red meat, full-fat dairy, and natural fats higher in the recommended dietary pattern and substantially raise daily protein recommendations. This stands in contrast to the more cautious approach taken by many other countries. For example, China’s 2022 dietary guidelines suggest a daily intake of 120–200 g of livestock and poultry meat, emphasizing lean cuts (17). Saturated fat represents a major proportion of fat in red meat, particularly in fatty tissue. Excessive saturated fat intake can raise LDL-C and promote atherosclerosis. Notably, saturated fats also serve as key substrates for hepatic TG synthesis. Although some studies have reported a link between red meat consumption and higher plasma TG levels (18), most existing research has focused on red meat as a broad category, leaving a gap in high-quality evidence specifically addressing the effect of beef, one of the most commonly consumed red meats, on TG levels. A small clinical trial (NCT04841460) conducted by Texas A&M University compared the lipid effects of low-fat (5%) and high-fat (25%) ground beef in men and found no short-term changes in total cholesterol (TC) or TG (19). However, such findings do not address key public health questions: Does the intake of unprocessed beef in daily diet lead to an increase in plasma TG levels? If not, is there a clear range or threshold of beef intake that is associated with an increase in TG? Is there a genetic-level regulation of the relationship between the two?

To address these knowledge gaps, the present study will conduct the following investigations. First, cross-sectional analyses will be performed utilizing the nationally representative National Health and Nutrition Examination Survey (NHANES) database from the United States and a regional population database from Urumqi, China, to validate the generalizability and specificity of the association between dietary patterns among adults in Eastern and Western cultural contexts. Subsequently, we will conduct Mendelian randomization using genetic instrumental variables associated with beef intake and TG levels to infer potential causal relationships from a genetic perspective. This approach will furnish higher-grade evidence for the “diet-lipid” relationship and establish a robust scientific foundation for public health policies and individualized nutritional interventions.

## 2 Materials and methods

### 2.1 Datasets and study population

NHANES is a nationally representative cross-sectional research program conducted by the Centers for Disease Control and Prevention (CDC) in the United States. Its primary objective is to systematically evaluate the health and nutritional status of the U.S. population through the collection of demographic information, biochemical indicators, and survey questionnaire data. This study utilized data from NHANES 2007–2018. Participants under 20 years of age (*n* = 25,651), those with missing TG measurements (*n* = 19,403), and those with incomplete unprocessed beef consumption data (*n* = 12,654) were excluded, resulting in a final analytic sample of 2,134 adults (Supplementary Figure S1). All data are publicly accessible from the NHANES website: https://wwwn.cdc.gov/nchs/nhanes/default.aspx.

The Chinese sample was derived from the Comprehensive Health Checkup Program, a public health initiative launched in 2016 by the Xinjiang Uygur Autonomous Region. This program provides free annual health examination, including blood tests, chest X-rays, abdominal color Doppler ultrasonography, and general physical examinations, to residents of all ethnicities and ages. From May to August 2024, our research team was involved in the program for individuals aged 18 years and above. A total of 5,539 participants completed the health examinations, and their results were subsequently matched and integrated into the database. Furthermore, we conducted comprehensive questionnaire surveys among 2,149 Han Chinese participants to collect data encompassing basic demographic characteristics, individual and family medical histories, smoking and alcohol consumption patterns, dietary habits, work, physical activity levels, and sleep quality. Therefore, the Chinese cohort analysis for this study was conducted utilizing data from the aforementioned 5,539 participants. After excluding individuals under 20 years of age (*n* = 8), those with missing TG data (*n* = 303), and those without recorded beef consumption data (*n* = 3,254), a final sample of 1,974 participants was included (Supplementary Figure S2).

### 2.2 Definition of beef intake

Beef intake data from NHANES were derived from the dietary interview component. The first day of intake was recorded in person at a mobile examination center, while the second day was collected via telephone 3–10 days later; the latter may be subject to notable subjective bias due to the absence of on-site verification and cross-checking by data collection personnel. Therefore, only the Day 1 dietary records were utilized for the relevant analyses. These interviews were conducted jointly by the U.S. Department of Agriculture (USDA) and the Department of Health and Human Services (DHHS). Within DHHS, the National Center for Health Statistics (NCHS) oversaw survey design and data collection, whereas the USDA’s Food Surveys Research Group (FSRG) managed data entry, coding, review, and database maintenance. Beef-containing foods were identified by matching dietary records with the WWEIA database and categorized into two groups: “Ground beef” and “Beef, excludes ground” (20).

Beef consumption data from the Urumqi dataset were obtained through questionnaire surveys. During data collection, only plain beef, corresponding to the unprocessed beef categories “Ground beef” and “Beef, excludes ground” in the NHANES dataset, was included to ensure consistent inclusion criteria across both datasets and enable reliable cross-population comparisons. In addition, information was collected on consumption frequency (per year/month/week/day), the number of eating occasions at each specified frequency, and the amount consumed per occasion (in grams). This allowed subsequent harmonization with the daily intake standardization used in the NHANES data for comparative analysis. First, reported frequencies were converted to a daily basis: yearly = 1/365, monthly = 1/30, weekly = 1/7, and daily = 1. Daily beef intake was then calculated as: Daily intake (g/d) = Normalized daily frequency × Number of eating occasions × Amount per occasion (g). This approach allowed all participants’ beef intake to be expressed in comparable units of g/d.

### 2.3 Covariates

For the NHANES component of this study, we collected baseline characteristics and laboratory measures for all participants. Baseline information included age, gender, race, education level, family income-to-poverty ratio, smoke status, alcohol status, exercise status, and self-reported histories of depression, hypertension, dyslipidemia, and diabetes. Laboratory measures comprised alanine aminotransferase (ALT), aspartate aminotransferase (AST), albumin, gamma-glutamyl transferase (GGT), total bilirubin (TBil), uric acid, serum creatinine (SCr), blood urea nitrogen (BUN), high-density lipoprotein cholesterol (HDL-C), LDL-C, TG, TC, glycated hemoglobin (HbA1c), and fasting plasma glucose (FPG).

We defined categorical variables as follows: Race is classified into Mexican American, other Hispanic, non-Hispanic white, non-Hispanic black, and other race. Education level is divided into <high school, high school, some college or AA degree, and college degree or above. Smoke status is classified as never, former, and current based on the responses to the questions “Have you smoked at least 100 cigarettes in your entire life?” and “Do you smoke cigarettes now?”. Alcohol status is categorized based on the average daily alcohol intake (g/d) calculated from the 24-h dietary recall data, and is divided into never (0 g/d), moderate (male: 0.1–24.9 g/d; female: 0.1–14.9 g/d), and heavy (male: ≥ 25.0 g/d; female: ≥ 15.0 g/d). Exercise status is classified based on the frequency of moderate and vigorous physical activity during leisure time per week, and is divided into inactivity (engaging in no physical activity), insufficient (<5 times of moderate activity or <3 times of vigorous activity per week), and active (≥5 times of moderate activity or ≥3 times of vigorous activity per week). For disease definitions, depression was indicated by a PHQ-9 score ≥ 10; hypertension by prior diagnosis, antihypertensive medication use, or measured blood pressure ≥ 130/80 mmHg; dyslipidemia by prior diagnosis, lipid-lowering medication use, or abnormal lipids (TC ≥ 6.2 mmol/L, LDL-C ≥ 4.1 mmol/L, or HDL-C < 1.0 mmol/L); and diabetes by prior diagnosis, glucose-lowering medication use, HbA1c ≥ 6.5%, or FPG ≥ 126 mg/dL.

For the Urumqi dataset, we collected baseline characteristics (age, gender, education level, family income, smoke status, alcohol status, exercise status, and histories of hypertension, dyslipidemia, and diabetes) and laboratory measures (ALT, AST, SCr, BUN, HDL-C, LDL-C, TG, TC, and FPG). Categorical variables were defined as follows: Education level in divided into <high school, high school, college degree or above; smoke status is classified into never, current and former based on responses to the questions: “Have you smoked at least 100 cigarettes (approximately 5 packs) in your lifetime?” and “Have you smoked in the past month?”; Alcohol status is divided into drinker and non-drinker according to the response to: “Have you consumed alcohol 12 times or more in the past 12 months?”; Exercise status is categorized as no exercise (engaging in no physical activity), insufficient exercise (light activity ≤ 15 h/week, or moderate activity ≤ 5 h/week, or vigorous activity ≤ 3 h/week), and regular exercise (light activity > 15 h/week, or moderate activity > 5 h/week, or vigorous activity > 3 h/week).

### 2.4 Mendelian randomization analysis

To investigate the potential causal relationship between beef consumption and TG, we performed two-sample Mendelian randomization analysis, using beef consumption as the exposure and TG as the outcome. Genetic instruments for beef consumption were obtained from Pirastu N (ebi-a-GCST90096901) (21), which included 241,092 participants of European ancestry. Instruments for TG were obtained from Borges CM (met-d-Total_TG) (22) and Richardson TG (ebi-a-GCST90092992) (23), comprising 115,078 and 115,082 European-ancestry participants. All genome-wide association study (GWAS) data are publicly accessible via the IEU OpenGWAS platform.^1^ To maximize the number of instrumental variables, single-nucleotide polymorphisms (SNPs) were selected at a significance threshold of *p* < 5 × 10^−6^, with clumping parameters set at 10,000 kb for distance and *r*^2^ < 0.001 for linkage disequilibrium (24). The primary causal estimate was derived using the inverse-variance weighted (IVW) method. With 95% confidence intervals (CI) derived as *β* ± 1.96 × SE. Sensitivity analyses were conducted using MR-Egger regression, weighted median, and weighted mod. Horizontal pleiotropy was evaluated via MR-PRESSO, and heterogeneity was examined using Cochran’s *Q* test. Detailed information on the GWAS summary data is provided in Supplementary Table S1.

### 2.5 Statistical analysis

In this study, statistical analysis was conducted as follows. Continuous variables were first assessed for normality using the Shapiro–Wilk test. If the data conformed to a normal distribution, they were presented as mean ± standard deviation and analyzed statistically using independent samples *t*-tests or analysis of variance (ANOVA). If the data did not conform to a normal distribution, they were expressed as median [p25, p75] and analyzed statistically using the Kruskal–Wallis *H* test. Categorical variables were expressed as frequency (percentage) and analyzed statistically using the Chi-square test or Fisher’s exact test. All statistical analyses were two-sided tests, and *p* < 0.05 was considered statistically significant. Due to the complex sampling survey design of NHANES, the corresponding analyses were conducted using weighted Kruskal–Wallis *H* tests and weighted Chi-square tests.

To improve clinical interpretability and ensure comparability across datasets, beef intake was standardized to increments of 100 g/d. Potential confounders (including demographic, behavioral, and clinical history variables) were first screened in both the NHANES and Urumqi datasets using univariable logistic regression (*p* < 0.05). Multivariable stepwise regression models were then constructed, with all models adjusted for total dietary energy intake to isolate the specific effect of beef consumption. For NHANES, Model 1 was unadjusted; Model 2 adjusted for age, gender, and race; and Model 3 additionally adjusted for education level, family income-to-poverty ratio, smoke status, alcohol status, exercise status, and medical histories of depression, hypertension, and diabetes. For the Urumqi dataset, Model 1 was unadjusted; Model 2 adjusted for age and gender; and Model 3 further adjusted for education level, family income, smoke status, alcohol status, exercise status, and medical histories of hypertension and diabetes. To assess model stability, sensitivity analyses were performed by categorizing beef consumption into quartiles. As TG levels were analyzed as a continuous variable, results are presented as *β* coefficients with 95%CI.

To explore potential nonlinear relationships, we fitted restricted cubic spline (RCS) models with four knots to examine the dose–response association between beef intake and hypertriglyceridemia risk. Additionally, we conducted stratified analyses to evaluate whether the observed associations were consistent across key subgroups. For the NHANES dataset, stratification variables included age (<60/≥60 years), gender, family income-to-poverty ratio (<1.3/1.3–3.5/≥3.5), smoke status (never/current/former), alcohol status (never/moderate/heavy), exercise status (inactive/active/insufficient), and histories of depression, hypertension, and diabetes. For the Urumqi dataset, subgroup analyses were performed by gender, smoke status (never/current/former), alcohol status, exercise status (inactive/active/insufficient), and histories of hypertension and diabetes.

All statistical analyses were performed in R (version 4.4.3). We used the following packages for specific analyses: baseline characteristics were summarized with “gtsummary,” RCS models were fitted using the “rms” package, Mendelian randomization analyses were conducted with “TwoSampleMR,” horizontal pleiotropy was assessed via “MR-PRESSO,” and meta-analyses were performed using the “meta” package.

## 3 Result

### 3.1 Participant characteristics

The NHANES dataset included 2,134 participants, with a median age of 48 years and a majority (56.11%) being male. The mean beef intake was 119.13 g/d, and intake quartiles were: Q1: 0.52 g/d-63.75 g/d, Q2: 63.75 g/d-100.50 g/d, Q3: 100.50 g/d-153.00 g/d, and Q4: 153.00 g/d-1088.00 g/d. The intergroup comparative analysis indicates that, relative to participants in the lowest quartile (Q1) of beef intake, those with higher beef consumption were predominantly male, younger in age, exhibited a higher proportion of non-Hispanic White, attained higher educational levels, and came from more affluent socioeconomic backgrounds. Moreover, although individuals with higher beef intake predominantly comprise non-smokers and non-drinkers, the proportion of those with a history of smoking and alcohol consumption is greater in the Q4 group compared to the Q1 group, and a higher proportion of this cohort maintains habitual physical activity. Regarding medical history, the Q4 cohort exhibited lower prevalence rates of depression, hypertension, and diabetes mellitus, but a higher prevalence of hyperlipidemia (with statistically significant intergroup differences observed only for age, gender, race, and household income, *p* < 0.05). In terms of biochemical indicators, although the median values of the main indicators in each group were all within the normal reference range, with the increase in beef intake, the median levels of ALT, AST, GGT, Uric acid, SCr, and BUN all showed a significant upward trend (all intergroup differences were *p* < 0.05). Regarding the lipid profile, individuals with higher beef consumption exhibited elevated levels of TC, TG, and LDL-C, accompanied by a reduction in HDL-C (with between-group differences achieving statistical significance for TC and HDL-C at *p* < 0.05). However, no significant differences were found in the blood glucose-related indicators (HbA1C and FPG) among the four groups (all *p* > 0.05) (Table 1).

**Table 1:** The baseline characteristics of NHANES participants.

Characteristic	Overall	Quartile 1 (0.52–63.75)	Quartile 2 (63.75–100.50)	Quartile 3 (100.50–153.00)	Quartile 4 (153.00–1088.00)	*p-*value
Age, years	48.00 (35.00, 61.00)	50.00 (38.00, 63.00)	50.00 (36.00, 65.00)	47.00 (33.00, 59.00)	46.00 (34.00, 58.00)	**<0.001**
Gender, *n* (%)						**<0.001**
Male	8.04 (56.11)	1.22 (37.28)	2.14 (51.42)	1.76 (56.97)	2.91 (76.82)	
Female	6.29 (43.89)	2.06 (62.72)	2.02 (48.58)	1.33 (43.03)	0.88 (23.18)	
Race, *n* (%)						**0.023**
Mexican American	1.49 (10.39)	0.43 (13.22)	0.39 (9.28)	0.37 (11.90)	0.29 (7.91)	
Other Hispanic	0.96 (6.70)	0.22 (6.85)	0.23 (5.53)	0.21 (6.98)	0.28 (7.64)	
Non-Hispanic White	9.73 (67.91)	2.00 (61.05)	2.99 (72.08)	2.10 (68.07)	2.62 (69.12)	
Non-Hispanic Black	1.34 (9.37)	0.36 (11.05)	0.39 (9.50)	0.26 (8.49)	0.32 (8.50)	
Other race	0.81 (5.64)	0.25 (7.82)	0.15 (3.62)	0.14 (4.56)	0.25 (6.83)	
Education, *n* (%)						**0.371**
<High school	2.56 (17.88)	0.72 (22.11)	0.70 (16.87)	0.41 (13.50)	0.71 (18.88)	
High school	3.19 (22.28)	0.75 (23.09)	0.90 (21.64)	0.77 (25.14)	0.75 (19.96)	
Some college or AA degree	4.26 (29.79)	0.91 (27.92)	1.26 (30.51)	1.00 (32.35)	1.08 (28.52)	
College graduate or above	4.30 (30.05)	0.88 (26.88)	1.28 (30.98)	0.89 (29.01)	1.23 (32.64)	
Family income-to-poverty ratio	3.04 (1.42, 5.00)	2.41 (1.22, 4.43)	3.11 (1.39, 5.00)	3.24 (1.48, 5.00)	3.50 (1.55, 5.00)	**0.004**
Smoke status, *n* (%)						**0.331**
Never	7.70 (53.78)	1.92 (58.71)	2.29 (55.04)	1.67 (54.24)	1.81 (47.75)	
Current	2.72 (19.00)	0.56 (17.18)	0.74 (18.02)	0.61 (19.96)	0.79 (20.86)	
Former	3.90 (27.22)	0.79 (24.11)	1.12 (26.93)	0.79 (25.80)	1.19 (31.40)	
Alcohol status, *n* (%)						**0.239**
Never	8.91 (62.19)	2.09 (63.91)	2.64 (63.53)	1.91 (61.88)	2.25 (59.49)	
Moderate	2.80 (19.58)	0.52 (16.06)	0.95 (22.86)	0.54 (17.60)	0.78 (20.66)	
Heavy	2.61 (18.23)	0.65 (20.03)	0.56 (13.61)	0.63 (20.52)	0.75 (19.86)	
Exercise, *n* (%)						**0.300**
Inactive	6.65 (46.42)	1.67 (50.97)	2.03 (48.80)	1.23 (39.77)	1.71 (45.29)	
Active	3.63 (25.35)	0.78 (24.07)	1.00 (24.17)	0.82 (26.54)	1.01 (26.77)	
Insufficient	4.04 (28.24)	0.81 (24.96)	1.12 (27.03)	1.04 (33.68)	1.06 (27.95)	
Medical history						
Depression, *n* (%)	1.02 (7.55)	0.30 (9.58)	0.22 (5.86)	0.29 (10.17)	0.19 (5.49)	0.053
Hypertension, *n* (%)	7.32 (51.15)	1.74 (53.22)	2.22 (53.57)	1.43 (46.36)	1.91 (50.60)	0.340
Dyslipidemia, *n* (%)	7.62 (53.22)	1.64 (50.03)	2.26 (54.52)	1.64 (53.06)	2.07 (54.69)	0.626
Diabetes, *n* (%)	2.16 (15.13)	0.47 (14.60)	0.80 (19.34)	0.43 (13.97)	0.45 (11.90)	0.090
Laboratory data^†^						
ALT, U/L	22.00 (16.00, 30.00)	20.00 (16.00, 27.00)	21.00 (16.00, 27.00)	22.00 (17.00, 30.00)	25.00 (18.00, 34.00)	**<0.001**
AST, U/L	23.00 (19.00, 27.00)	22.00 (18.00, 27.00)	22.00 (19.00, 26.00)	23.00 (19.00, 27.00)	24.00 (21.00, 28.00)	**<0.001**
Albumin, g/L	43.00 (41.00, 45.00)	42.00 (40.00, 44.00)	43.00 (41.00, 45.00)	43.00 (41.00, 45.00)	43.00 (41.00, 45.00)	**0.019**
GGT, U/L	20.00 (15.00, 31.00)	20.00 (14.00, 31.00)	19.00 (15.00, 30.00)	20.00 (15.00, 30.00)	22.00 (17.00, 34.00)	**0.002**
TBil, umol/L	11.97 (8.55, 13.68)	10.26 (8.55, 13.68)	11.97 (8.55, 15.39)	11.97 (8.55, 13.68)	11.97 (8.55, 13.68)	**0.011**
Uric acid, umol/L	327.10 (273.60, 386.60)	309.30 (255.80, 362.80)	327.10 (267.70, 380.70)	327.10 (267.70, 392.60)	356.90 (303.30, 404.50)	**<0.001**
SCr, umol/L	76.02 (63.65, 89.28)	72.49 (59.23, 83.10)	76.02 (63.65, 89.28)	75.14 (63.65, 87.52)	80.44 (70.72, 91.05)	**<0.001**
BUN, mmol/L	5.00 (3.93, 6.07)	4.64 (3.57, 5.71)	5.00 (3.93, 6.07)	5.00 (3.93, 6.07)	5.00 (4.28, 6.43)	**<0.001**
TC, mmol/L	4.91 (4.32, 5.64)	4.97 (4.32, 5.69)	4.73 (4.24, 5.59)	4.91 (4.34, 5.69)	5.07 (4.45, 5.69)	**0.020**
TG, mmol/L	1.22 (0.85, 1.81)	1.16 (0.82, 1.73)	1.21 (0.84, 1.75)	1.21 (0.88, 1.86)	1.30 (0.88, 1.95)	0.058
HDL-C, mmol/L	1.29 (1.09, 1.55)	1.37 (1.14, 1.63)	1.29 (1.09, 1.60)	1.29 (1.06, 1.53)	1.24 (1.03, 1.53)	**0.005**
LDL-C, mmol/L	2.95 (2.38, 3.52)	2.95 (2.41, 3.49)	2.85 (2.30, 3.41)	2.97 (2.41, 3.57)	3.08 (2.43, 3.62)	0.078
HbA1C, %	5.50 (5.20, 5.80)	5.50 (5.30, 5.80)	5.50 (5.30, 5.90)	5.40 (5.20, 5.70)	5.50 (5.20, 5.80)	0.086
FPG, mmol/L	5.61 (5.22, 6.16)	5.55 (5.22, 6.11)	5.61 (5.22, 6.16)	5.66 (5.22, 6.11)	5.72 (5.33, 6.16)	0.596

Data were presented as median [p25, p75] or *n* [In millions] (%). The weighted Kruskal–Wallis *H* test for continuous variables, and the weighted Chi-square test was employed for categorical variables to assess differences in the descriptive analyses. ^†^Presence of missing values. Bold text indicates statistical significance. ALT, alanine aminotransferase; AST, aspartate aminotransferase; GGT, *γ*-glutamyl transaminase; TBil, total bilirubin; Scr, serum creatinine; BUN, blood urea nitrogen; TC, total cholesterol; TG, triglyceride; HDL-C, high-density lipoprotein cholesterol; LDL-C, low-density lipoprotein cholesterol; FPG, fasting plasma glucose.

The Urumqi dataset included 1,974 participants (57.80% female), with a median age of 66 years. Mean beef intake was 80.54 g/d, distributed across quartiles as follows: Q1: 0.36 g/d-21.43 g/d, Q2: 21.43 g/d-74.29 g/d, Q3: 74.29 g/d-130 g/d, and Q4: 130 g/d-450 g/d. In partial alignment with trends observed in previous NHANES populations, the Urumqi dataset indicates that individuals in the Q4 group, compared to those in Q1, were predominantly male and exhibited higher educational attainment, yet demonstrated higher proportions of current smokers, alcohol consumers, and physically inactive individuals. In terms of disease prevalence, the high-intake group showing lower hypertension but higher dyslipidemia and diabetes rates. Regarding biochemical indicators, ALT and SCr levels increased with beef intake, whereas AST and BUN tended to decrease (*p* < 0.05 for ALT, AST, and SCr). Lipid profiles displayed a pattern divergent from that of the NHANES population: higher beef intake was associated with lower LDL-C, and the Q4 group had the lowest TC and HDL-C levels (*p* < 0.05 for TC, HDL-C, and LDL-C). Additionally, FPG level was also significantly higher in Q4 than in Q1 (*p* < 0.05) (Supplementary Table S2).

### 3.2 Association between beef intake and TG

**Figure 1:**
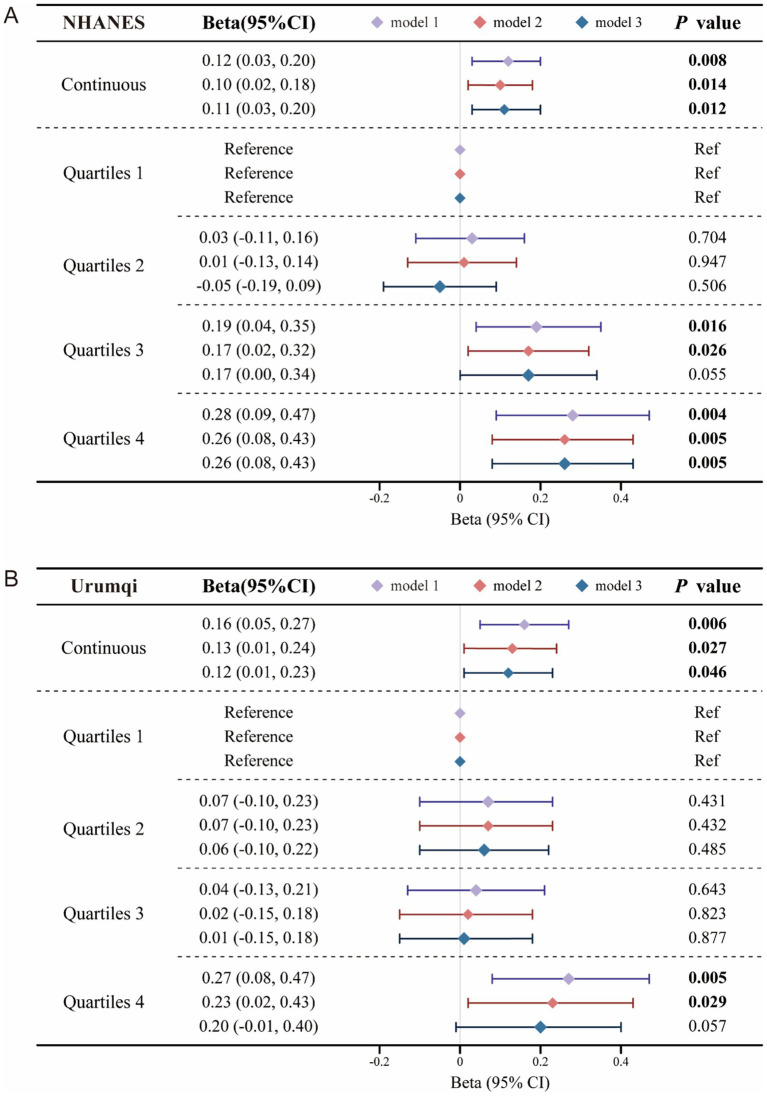
Multivariable regression analysis of beef intake and triglyceride levels. **(A)** Results from the NHANES cohort. **(B)** Results from the Urumqi cohort. Bold text indicates statistical significance.

In the NHANES dataset, each 100 g/d increase in beef intake was associated with a 0.12 mmol/L rise in TG levels in the unadjusted model (95%CI: 0.03–0.20, *p* = 0.008). After full adjustment for age, gender, race, and other covariates, the association remained significant, though slightly attenuated (*β* = 0.11, 95%CI: 0.03–0.20, *p* = 0.012). Sensitivity analysis using intake quartiles showed that the highest quartile (Q4) was independently associated with a 0.26 mmol/L increase in TG compared with the lowest quartile (Q1) (95% CI: 0.08–0.43, *p* = 0.005). In the Urumqi dataset, the fully adjusted model also indicated that each 100 g/d increment in beef intake was related to a 0.12 mmol/L increase in TG (95% CI: 0.01–0.23, *p* = 0.046), aligning with the NHANES trend. However, further sensitivity analysis in this cohort suggested limited stability in the fully adjusted model (*β* = 0.20, 95% CI: −0.01 to 0.40, *p* = 0.057) (Supplementary Tables S3–S6, Figure 1).

We used RCS models to examine the dose–response relationship between beef consumption and hypertriglyceridemia risk. No significant nonlinear association was observed in either the NHANES (*p* for nonlinearity = 0.1042) or Urumqi dataset (*p* for nonlinearity = 0.2471), suggesting that a linear model best described the relationship across the intake ranges studied. However, the RCS results also indicate that in both the U.S. and Chinese adult populations, when daily beef consumption exceeds approximately 100 g, the risk of hypertriglyceridemia shifts from suppression (OR < 1) to promotion (OR > 1). Specifically, the risk begins to increase in U.S. adults when intake exceeds 101.3 g/d, plateauing around 200 g/d. In contrast, the risk exhibits a nearly linear upward trend in Chinese adults once intake surpasses 111.8 g/d (Supplementary Figure S3).

To determine whether the association between beef intake and TG levels varies across population subgroups, we performed subgroup analyses in both the U.S. (NHANES) and Chinese (Urumqi) cohorts. The analysis results indicated that the positive association between beef intake and TG levels was consistent across subgroups defined by age, gender, household income, smoke status, alcohol status, exercise, and the presence of depression, hypertension, and diabetes (all *p* for interaction > 0.05), suggesting that no significant population specificity was observed for this association (Supplementary Figure S4).

### 3.3 Mendelian randomization analysis

**Figure 2:**
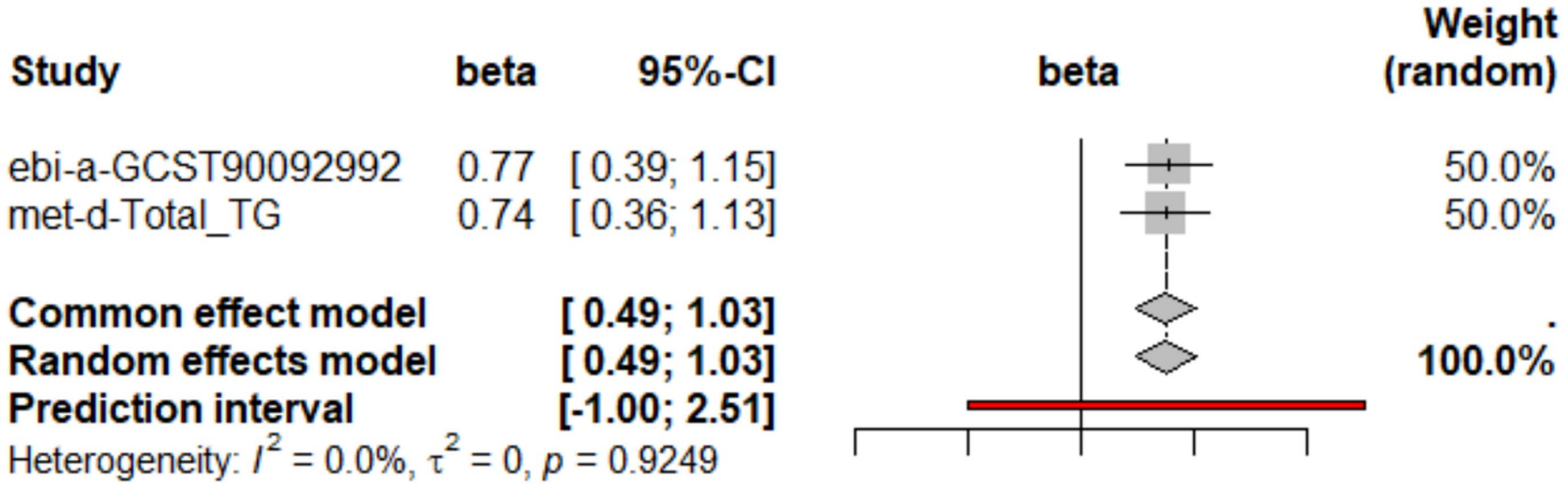
Meta-analysis of genetic associations between beef intake and TG levels. Data sources: ebi-a-GCST90092992 and met-d-Total_TG.

To evaluate the potential causal effect of beef intake on TG levels, we performed a two-sample Mendelian randomization analysis. The primary IVW method indicated a positive causal relationship, where genetically predicted higher beef consumption was associated with elevated TG levels. This finding was consistent across two independent genetic instrument sources: ebi-a-GCST90096901 (*β* = 0.769, 95% CI: 0.387–1.151, *p* = 0.001) and met-d-Total_TG (*β* = 0.743, 95% CI: 0.361–1.125, *p* = 0.001). Sensitivity analyses supported the robustness of these results: while MR-Egger regression showed a directionally inconsistent estimate (*β* < 0, *p* > 0.05), its intercept test revealed no significant horizontal pleiotropy (*p* = 0.124 and 0.111, respectively). The weighted median method yielded estimates in line with the IVW results (*β* ≈ 0.613–0.616, *p* < 0.05). Furthermore, no significant heterogeneity or outlier influence was detected by Cochran’s *Q* test or MR-PRESSO (all *p* > 0.05), and leave-one-out analysis as well as funnel plots confirmed model reliability (Supplementary Table S7, Supplementary Figures S5–S8). A meta-analysis combining the two IVW estimates yielded a pooled *β* of 0.756 (95% CI: 0.486–1.026, *p* < 0.001) with no evidence of heterogeneity (*I*^2^ = 0%, *τ*^2^ = 0, *p* = 0.9249) (Figure 2). Converting this value to an odds ratio reveals that beef consumption increases the risk of hypertriglyceridemia by approximately 2.1-fold (pooled OR = 2.13, 95% CI: 1.63–2.79).

## 4 Discussion

This study systematically evaluated the relationship between beef consumption and TG levels using both cross-sectional and Mendelian randomization approaches. The main findings are as follows: (1) cross-sectional analyses in U.S. and Chinese adults consistently showed a positive association between higher unprocessed beef intake and elevated TG levels; (2) RCS analysis revealed a significant upward trend in the risk of hypertriglyceridemia when daily beef intake exceeded approximately 100 g, indicating a potential inflection point in the dose–response relationship; and (3) Mendelian randomization provided genetic evidence supporting a causal role of beef intake in raising TG levels, as demonstrated by inverse-variance weighted estimates.

### 4.1 Beef consumption patterns across population subgroups

Our baseline analyses revealed distinct profiles of beef consumers between the NHANES (U.S.) and Urumqi (China) adults, shaped by differing demographic, socioeconomic, and cultural contexts. In the younger, ethnically diverse U.S. cohort, high beef intake was more common among younger males and non-Hispanic White people with higher household incomes—a pattern consistent with Western studies linking red meat consumption to male sex and higher socioeconomic status (25, 26). In the older Urumqi population, high consumers were also predominantly male, but higher educational attainment emerged as a key characteristic, possibly reflecting how greater nutritional awareness and economic capacity promote the consumption of quality animal protein in this setting (27). The divergence in consumption motivations—potentially driven more by culture and habit in the U.S. and by awareness and economic factors in Urumqi, China—is crucial for understanding dietary behavior differences between the two regions.

Health behaviors also differed between the two groups. Within the NHANES cohort, although no statistically significant differences were observed across groups in terms of smoking, alcohol consumption, physical activity, or prevalence of various diseases, certain trends were noticeable. Specifically, as beef intake increased, the proportion of non-smokers and non-drinkers gradually declined, while the percentage of individuals with adequate physical activity correspondingly rose. Regarding disease prevalence, no clear graded trends were detected for depression, hypertension, hyperlipidemia, or diabetes. However, compared to the Q1 group, the high-intake group exhibited an elevated prevalence of hyperlipidemia. Overall, despite a degree of health consciousness regarding physical activity among the U.S. population, the coexistence of high meat consumption, smoking, and alcohol use contributes to a significantly increased risk of hyperlipidemia (28, 29). Conversely, data from Urumqi present a different pattern: high beef consumers exhibited significantly higher proportions of current smokers and alcohol drinkers, along with an extremely high rate of physical inactivity (approximately 75%). This outlines a more synergistic clustering of risk behaviors, where high beef intake may coincide with other unhealthy habits such as smoking, alcohol use, and sedentary behavior, collectively forming a risk cluster for metabolic disease (30). Such aggregation of risk behaviors may partly explain why, in the Urumqi population, higher beef consumption is associated with a trend toward greater diabetes prevalence and elevated fasting blood glucose levels.

Biomarker profiles further highlighted metabolic differences. While higher beef intake was associated with increased ALT and SCr in both cohorts, suggesting potential metabolic burden. In the U.S. population, it correlated with an atherogenic pattern (higher TG, lower HDL-C). In the Chinese cohort, the highest intake group showed the lowest levels of TC and HDL-C, alongside a downward trend in LDL-C. These contrasting lipid profiles suggest that factors such as genetic background, overall dietary structure (e.g., dietary fiber intake, consumption of other fats), or cooking methods (e.g., use of oils in Chinese cuisine) may substantially modulate or even reverse the potential impact of saturated fats from beef on blood lipids (31–33).

### 4.2 A dose–response relationship between beef intake and TG levels

This study identified a significant positive association between beef consumption and serum TG levels, providing important epidemiological evidence for understanding dietary impacts on lipid metabolism. However, a thorough analysis of the underlying mechanisms behind this association, especially the trends indicated by the RCS analysis that, although not reaching traditional statistical significance, are still worthy of attention, reveals that it is not a simple linear relationship but may exhibit a dose-dependent characteristic with public health significance. Specifically, the data suggest that within a relatively low intake range (for instance, in the case of NHANES data, when daily intake is less than approximately 100 g), an increase in beef consumption may not promote an elevation in TG and might even have a neutral or subtly positive impact on the lipid profile due to the effects of other nutrients; however, once the intake exceeds a certain threshold, the negative effects of its specific risk components take the lead, driving up TG levels.

At lower intake levels (<100 g/d), beef consumption may have a neutral or even modestly protective effect on TG levels. Beef provides high-quality protein, highly bioavailable heme iron, zinc, selenium, and B vitamins—notably B12—all of which contribute to energy metabolism and cardiovascular health (34). Adequate protein can promote satiety and help maintain lean mass, indirectly supporting metabolic balance (35). Zinc and selenium act as cofactors for antioxidant enzymes, potentially reducing oxidative stress linked to dyslipidemia (36). Furthermore, beef, especially grass-fed varieties, contains conjugated linoleic acid (CLA), which in cellular and animal models has shown favorable effects on body composition and lipid metabolism (37, 38). Thus, at low to moderate intake levels, the beneficial nutrients may partially counteract or mask the adverse effects of saturated fat, leading to a plateau in the TG response curve. This is evidenced by either minimal change in TG levels with slight increases in very low intake (as seen in the initial segment of the NHANES RCS curve) or a mild decline (reflected in the shallow downward trend of the mid-section of the Urumqi curve), collectively suggesting the existence of a potential “safety window” or “equilibrium point.”

When daily beef consumption consistently exceeds approximately 100 grams, the cumulative effects of several dietary components likely explain the positive association with elevated TG observed in our study. First, the high saturated fat content—particularly palmitic acid in fattier cuts—plays a central role. Saturated fatty acids can stimulate hepatic SREBP-1c, promoting *de novo* TG synthesis and increasing circulating TG-rich lipoproteins such as VLDL (39, 40). Second, the contributory role of dietary cholesterol should be noted. Although the impact of dietary cholesterol on plasma total cholesterol exhibits considerable interindividual variation, and recent guidelines have relaxed restrictions on dietary cholesterol intake, high-dose consumption (often accompanying high beef intake) may still elevate TG levels in certain populations, particularly cholesterol-sensitive individuals (41). Finally, high-temperature cooking methods (e.g., grilling or frying) generate compounds like heterocyclic amines and polycyclic aromatic hydrocarbons, which may contribute to dyslipidemia by promoting systemic inflammation and oxidative stress (42, 43). At high consumption levels, exposure to these cooking-derived by-products increases correspondingly, potentially further disrupting lipid metabolism.

Overall, the effect of beef consumption on TG levels appears to reflect a dynamic balance between nutrients with favorable effects and harmful constituents, which shifts across intake levels. At lower amounts, beneficial components such as high-quality protein, minerals, and certain fatty acids may predominate. However, once intake surpasses an individual’s metabolic capacity, approximately above 100 g/d based on our observations, the adverse contributions from saturated fats, dietary cholesterol, and cooking-derived compounds become dominant, collectively elevating TG concentrations. This dose-dependent pattern highlights that blanket dietary advice on red meat may be inadequate. Our findings support moderating beef intake within a reasonable range, such as the 120–200 g/d recommended for poultry and livestock meat in the *Dietary Guidelines for Chinese Residents (2022)*, preferably choosing lean cuts, to help minimize its adverse impact on blood lipids.

### 4.3 Genetically supported causal link between beef intake and TG

Our Mendelian randomization analysis provides genetically informed evidence for a causal effect of beef consumption on elevated TG. Using the inverse-variance weighted method as the primary approach, each unit increase in genetically predicted beef intake was associated with a 0.756 mmol/L rise in plasma TG levels (95% CI: 0.486–1.026, *p* < 0.001), a finding supported by multiple sensitivity analyses. These results move beyond the limitations of observational studies, suggesting that lifelong dietary preferences influenced by genetic background may themselves contribute to dyslipidemia. Notably, the genetic variants used as instruments are not random markers; they are largely located in or near genes involved in appetite regulation, lipid metabolism, and energy homeostasis. Together, these genes form a molecular bridge linking dietary behavior to metabolic outcomes, primarily through three distinct pathways.

Genes that influence appetite and food preference—particularly for energy-dense foods—represent one key pathway. A prominent example is the *FTO* (fat mass and obesity-associated) gene. GWAS have consistently linked specific *FTO* variants (e.g., rs1421085) not only to obesity risk but also to a higher genetic predisposition for red meat and saturated fat intake (44, 45). Mechanistically, *FTO* is expressed in central regions such as the hypothalamus, where it helps modulate leptin and ghrelin signaling, thereby affecting energy balance and food-reward response (46, 47). Carriers of risk alleles may therefore show a stronger preference for energy-dense foods like beef. When this genetic predisposition leads to habitual high beef consumption, the resulting sustained load of saturated fats supplies abundant substrate for hepatic TG synthesis, ultimately raising plasma TG levels. In this way, *FTO* acts as a dual-influence gene, promoting both the desire to consume more beef and a metabolic tendency toward lipid storage, thereby contributing to hypertriglyceridemia.

Genetic variation in genes directly involved in lipid handling influences how the body responds to high-fat foods such as beef. *APOA5*, for example, encodes a protein that is a critical negative regulator of plasma TG. Loss-of-function variants impair the activation of lipoprotein lipase (LPL) and reduce clearance of TG-rich lipoproteins (48, 49). When high beef consumption increases the flux of dietary saturated fat and cholesterol—raising chylomicron production and hepatic VLDL secretion—individuals with reduced *APOA5* activity may be less able to clear the resulting TG load, leading to higher circulating TG levels. Similarly, the *LPL* gene encodes the rate-limiting enzyme for TG hydrolysis (50). Gain-of-function variants in *LPL* (e.g., rs1121923, rs258) are associated with significantly lower plasma TG, reflecting an enhanced capacity to hydrolyze TG from chylomicrons and VLDL (51). Thus, an individual’s genetically determined *LPL* activity fundamentally shapes their ability to manage the postprandial TG rise after a high-fat meal, including one rich in beef.

Genetic variation in energy-sensing and fat-storage pathways influences how ingested energy, particularly from high-fat foods like beef, is partitioned. The *GCKR* gene encodes the glucokinase regulator; the common variant rs1260326 alters hepatic glucokinase activity and glucose metabolism, predisposing carriers to a state of enhanced hepatic lipogenesis (52). Individuals carrying specific alleles may exhibit a “high TG synthesis readiness state,” potentially through enhanced hepatic glycolytic flux and increased substrate availability for *de novo* lipogenesis. In this setting, high beef intake—which supplies both exogenous fat and protein that can be converted to glucose—may synergistically boost endogenous TG synthesis. Variants in adipocyte-related genes such as *PPARG* can also modulate fasting TG levels by affecting adipose tissue lipid-storage capacity (53). Furthermore, cholesteryl ester transfer protein (CETP) mediates the exchange of TG from VLDL with cholesteryl esters from HDL. Elevated CETP activity promotes TG enrichment of HDL and LDL, leading to their faster clearance and ultimately to lower HDL-C and higher remnant TG levels (54). Notably, saturated fats in beef have been shown to upregulate CETP activity (55). Thus, individuals with genetic variants that confer higher basal CETP activity may experience a compounded, diet-induced dysregulation of lipid exchange when consuming large amounts of beef, collectively.

### 4.4 Limitation

Despite the multi-method design employed in this study, incorporating cross-population analyses from Chinese and U.S. cohorts and supporting causal inference via Mendelian randomization, several limitations should be acknowledged. First, substantial differences in demographic characteristics, dietary cultures, and baseline health profiles between the two populations may affect the generalizability and direct comparability of the results. Second, dietary data relied on self-reported recall, which is susceptible to recall bias and may reduce the precision of intake estimates. Furthermore, although a significant genetic correlation was identified, functional evidence is still needed to clarify how specific genes influence beef preference and subsequent metabolic pathways. Translating these statistical associations into well-defined biological mechanisms will require further research.

## 5 Conclusion

This study systematically evaluated the relationship between beef consumption and serum TG levels by integrating cross-sectional analyses with Mendelian randomization. We observed a consistent, positive dose–response relationship between higher beef intake and elevated TG in both U.S. (NHANES) and Chinese (Urumqi) populations. Using two-sample Mendelian randomization, we further obtained genetic evidence for a causal effect, with genetically predicted higher beef intake associated with increased TG levels (*β* = 0.756). Mechanistically, this link may involve genes related to appetite regulation (e.g., *FTO*) and lipid metabolism (e.g., *APOA5*, *LPL*). By converging evidence from three complementary perspectives, observational association, causal inference, and biological mechanism, this study identifies beef intake as a modifiable dietary factor influencing TG, supporting more nuanced guidance on red meat consumption within personalized nutrition and cardiovascular disease prevention strategies.

## Data Availability

The original contributions presented in the study are included in the article/Supplementary material, further inquiries can be directed to the corresponding author.

## Footnotes

1
https://gwas.mrcieu.ac.uk/
