# Supplementary material for: High unprocessed beef intake (>100 g/d) linked to elevated triglyceride levels: evidence from cross-sectional and Mendelian randomization in Chinese and American adult populations
Source: Front Nutr. 2026 May 14;13:1790394. doi: 10.3389/fnut.2026.1790394 (PMC13215848; doi:10.3389/fnut.2026.1790394)
Supplement: Supplementary file 1 [file Table_1.docx]

Supplement Materials for

**High unprocessed beef intake (> 100 g/d) linked to elevated triglyceride levels: evidence from cross-sectional and mendelian randomization in Chinese and American adult populations**

Ziyu Yi^1^, Shuying Ding^1^, Xiangyang Liu^1^, Shuai Liu^1^, Zhenyan Fu^1^

(^1^First Affiliated Hospital of Xinjiang Medical University, Urumqi, Xinjiang 830054, China)

This file includes:

Figures. S1 to S8

Tables. S1 to S7


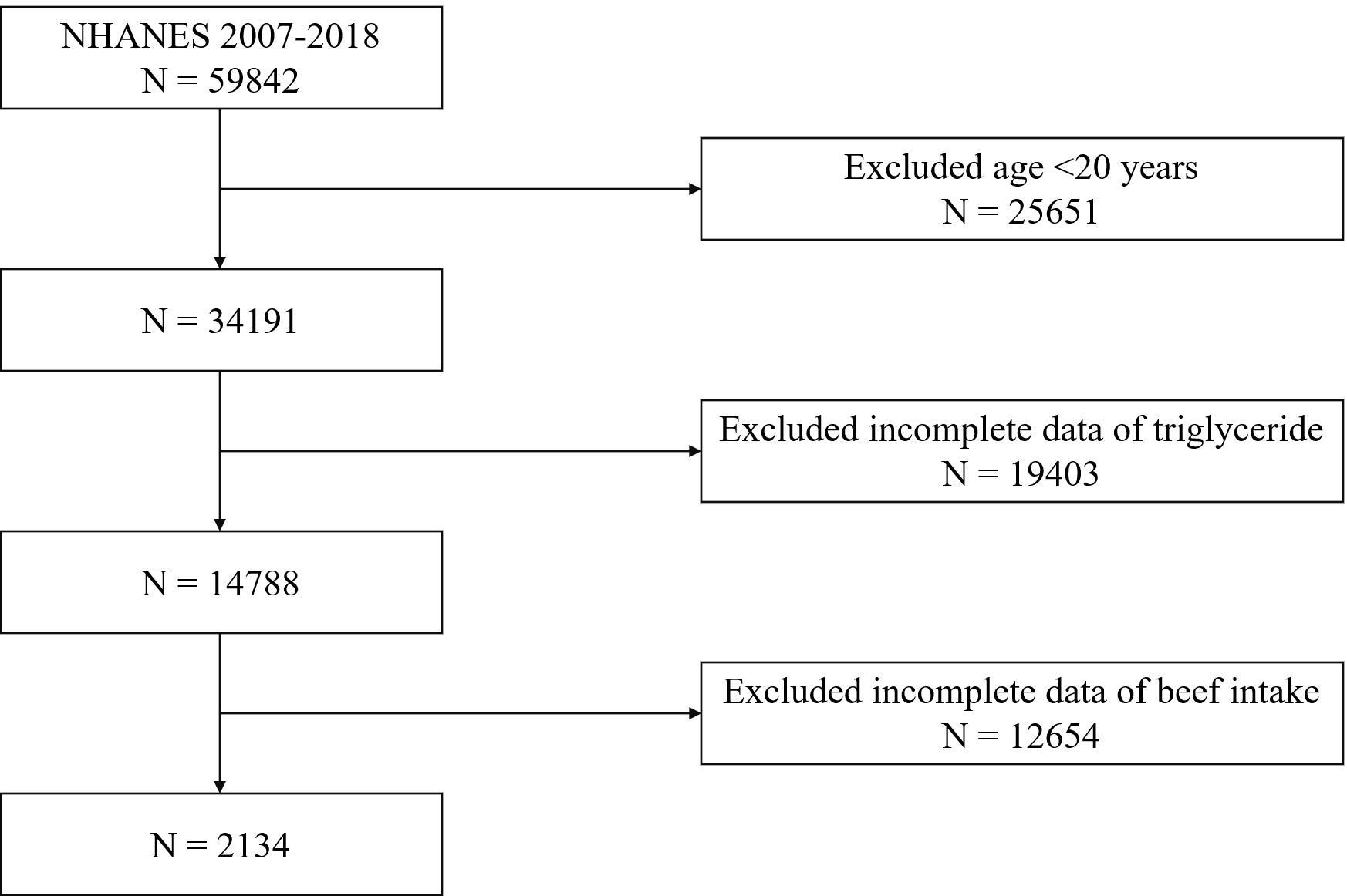


**Figure S1** Flowchart of participant selection from NHANES 2007-2018.


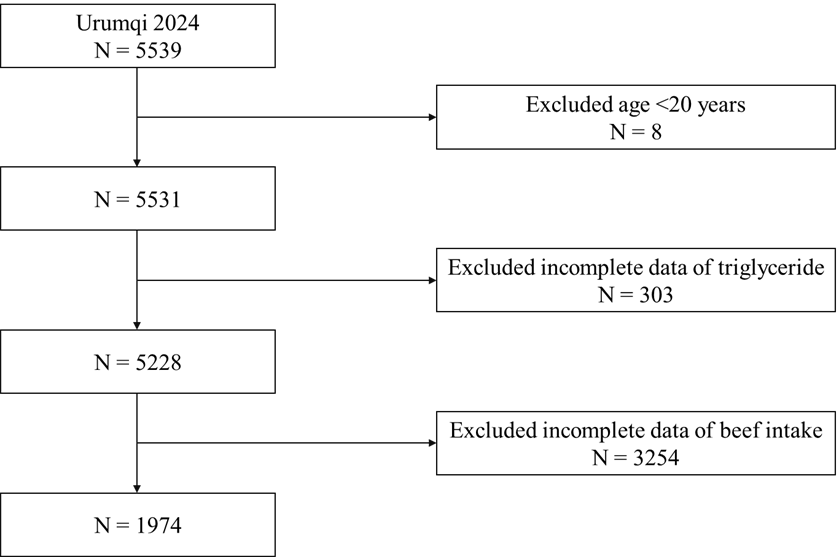


**Figure S2** Flowchart of participant selection from the Chinese cohort study (Urumqi, 2024).


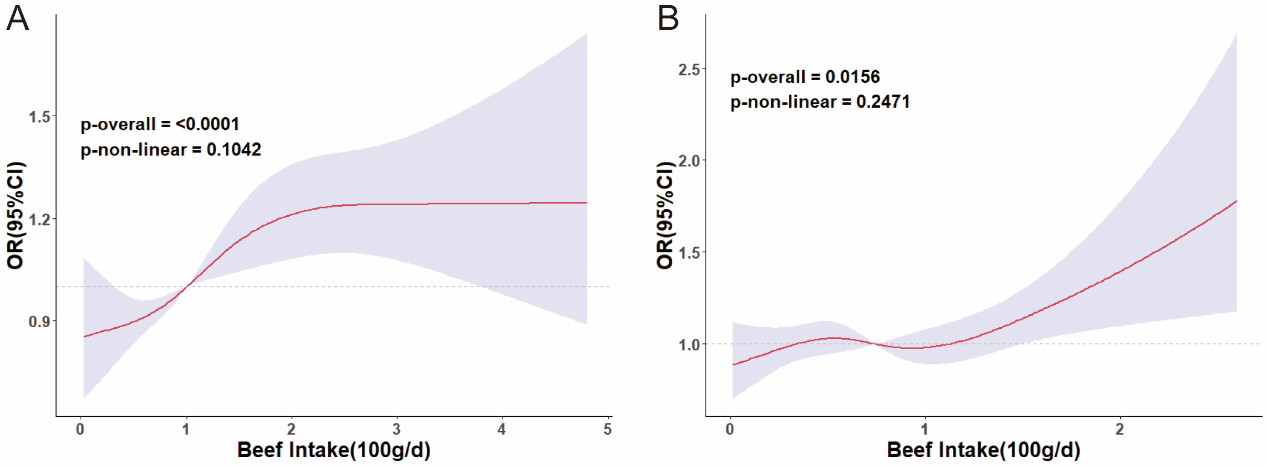


**Figure S3** Nonlinear associations between beef intake and triglyceride levels by RCS analysis. (A) Results from the NHANES cohort. (B) Results from the Urumqi cohort.

**
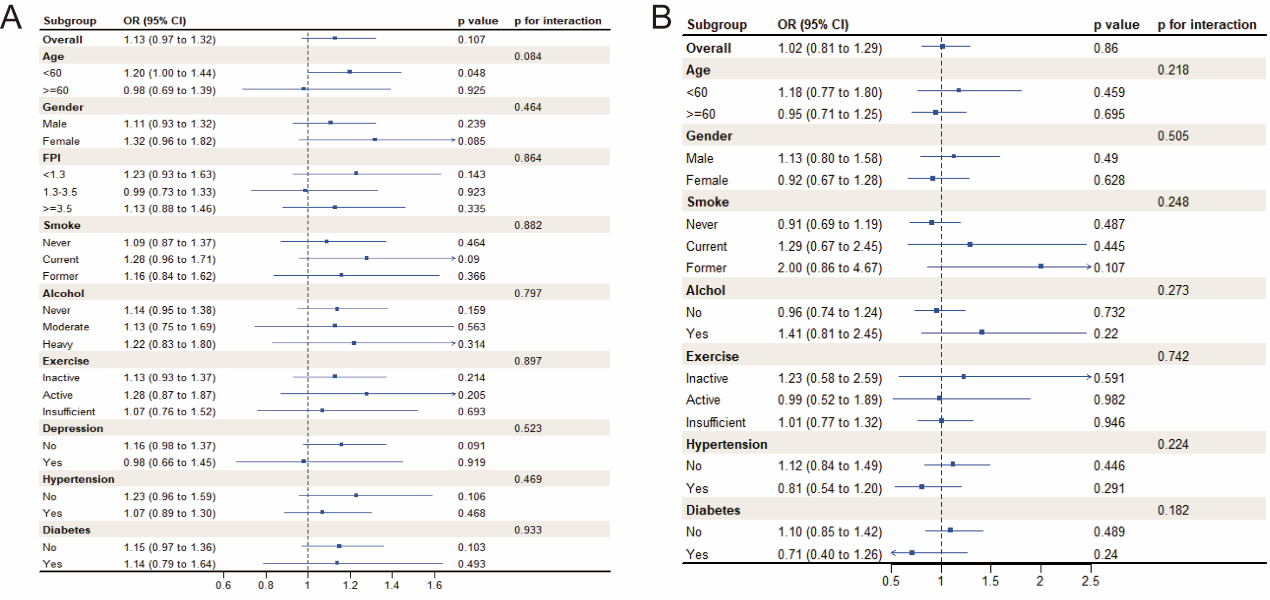
**

**Figure S4** Stratified analyses of the associations between beef intake and triglyceride levels. (A) Results from the NHANES cohort. (B) Results from the Urumqi cohort.


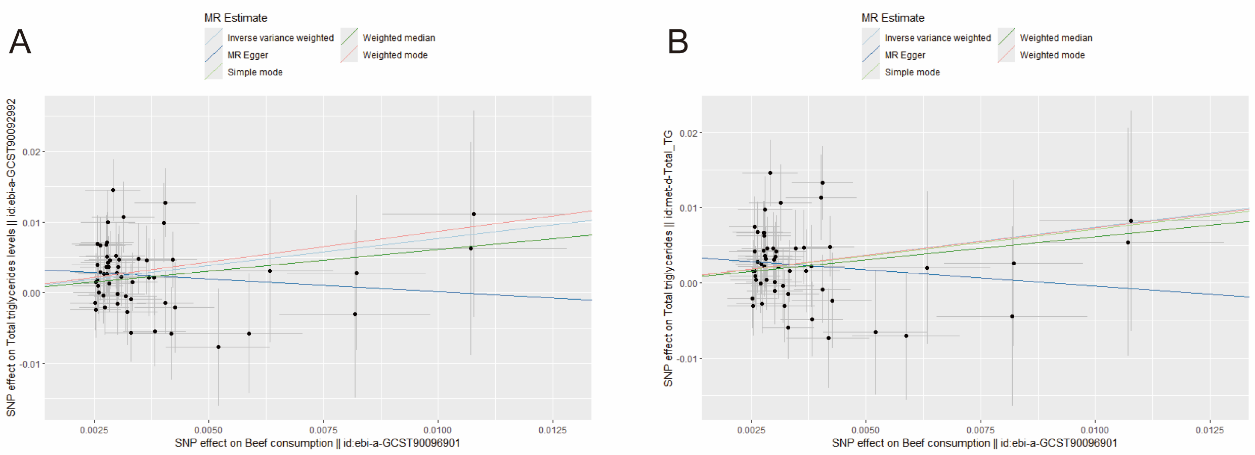


**Figure S5** MR analysis scatter plot of beef intake on triglyceride levels. (A) ebi-a-GCST90092992; (B) met-d-Total_TG.


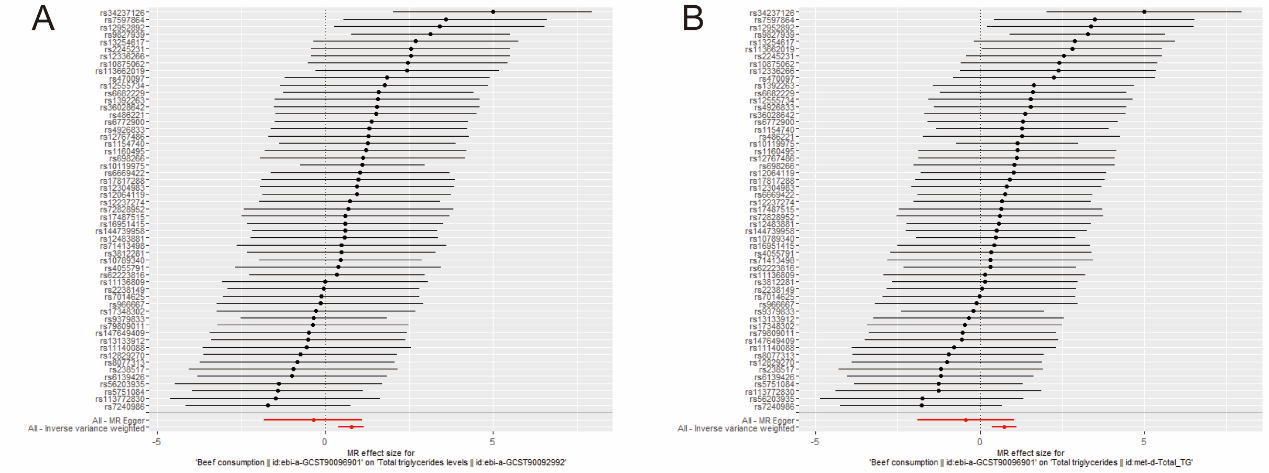


**Figure S6** MR analysis forest plot of beef intake on triglyceride levels. (A) ebi-a-GCST90092992; (B) met-d-Total_TG.


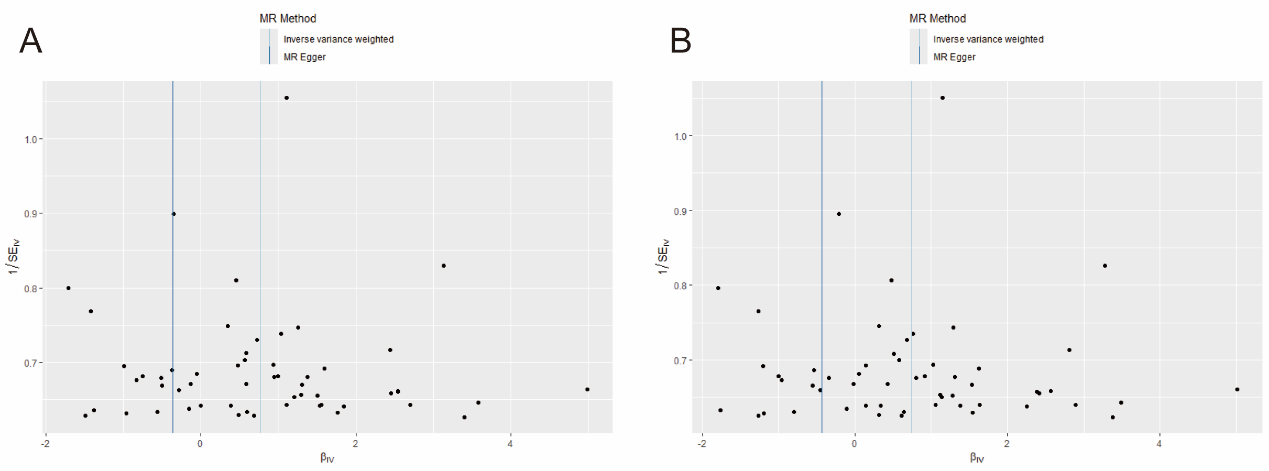


**Figure S7** MR analysis funnel plot of beef intake on triglyceride levels. (A) ebi-a-GCST90092992; (B) met-d-Total_TG.


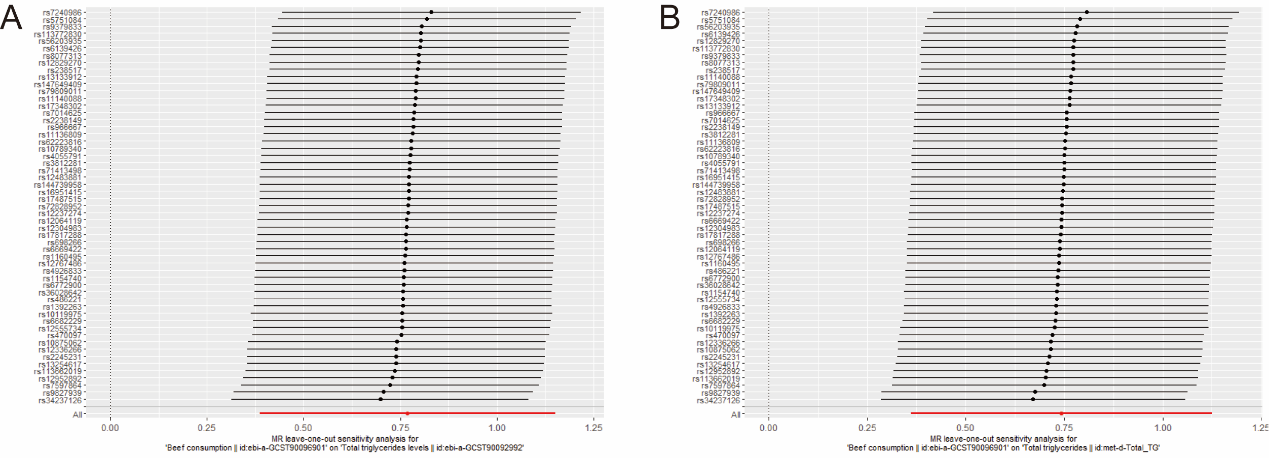


**Figure S8** MR analysis leave-one-out analysis of beef intake on triglyceride levels. (A) ebi-a-GCST90092992; (B) met-d-Total_TG.

**Table S1** Characteristics of GWAS used in the MR analysis.

| **Items** | **GWAS ID** | **Consortium** | **Sample size** | **Year** | **Population** |
| --- | --- | --- | --- | --- | --- |
| **Exposure** |  |  |  |  |  |
| Beef Intake | ebi-a-GCST90096901 | IEU | 241,092 | 2022 | European |
| **Outcomes** |  |  |  |  |  |
| Triglyceride | ebi-a-GCST90092992 | IEU | 115,082 | 2022 | European |
|  | met-d-Total_TG | IEU | 115,078 | 2020 | European |

GWAS: Genome-wide association study.

**Table S2** The baseline characteristics of Urumqi participants

| **Characteristic** | **Overall** | **Quartile 1 （0.36-21.43）** | **Quartile 2 （21.43-74.29）** | **Quartile 3 （74.29-130）** | **Quartile 4 （130-450）** | ***P* value** |
| --- | --- | --- | --- | --- | --- | --- |
| **Age, years** | 66.00(59.00,72.00) | 66.00(60.00,73.00) | 66.00(59.00,72.00) | 66.00(59.00,71.00) | 66.00(60.00,71.00) | 0.241 |
| **Gender, n (%)** |  |  |  |  |  | **<0.001** |
| Male | 0.83(42.20) | 0.19(37.50) | 0.17(28.22) | 0.21(37.05) | 0.27(85.44) |  |
| Female | 1.14(57.80) | 0.31(62.50) | 0.44(71.78) | 0.35(62.95) | 0.05(14.56) |  |
| **Education, n (%)** |  |  |  |  |  | **0.002** |
| ＜High School | 1.07(53.95) | 0.29(59.27) | 0.34(56.27) | 0.28(50.18) | 0.15(47.78) |  |
| High School | 0.41(20.97) | 0.09(18.35) | 0.12(19.14) | 0.14(25.36) | 0.07(20.89) |  |
| College graduate or above | 0.49(25.08) | 0.11(22.38) | 0.15(24.59) | 0.14(24.46) | 0.10(31.33) |  |
| **Family income, thousand** | 60.00 (40.00,96.00) | 60.00 (36.00,96.00) | 60.00 (40.00,100.00) | 60.00 (40.00,96.00) | 60.00 (48.00,96.00) | 0.086 |
| **Smoke status, n (%)** |  |  |  |  |  | **<0.001** |
| Never | 1.58(80.14) | 0.40(81.25) | 0.53(87.62) | 0.47(83.63) | 0.18(57.91) |  |
| Current | 0.22(11.09) | 0.05(9.68) | 0.04(7.26) | 0.04(7.91) | 0.08(26.27) |  |
| Former | 0.17(8.76) | 0.05(9.07) | 0.03(5.12) | 0.05(8.45) | 0.05(15.82) |  |
| **Alcohol status, n (%)** | 0.31(15.75) | 0.05(9.88) | 0.07(11.39) | 0.07(13.31) | 0.12(37.66) | **<0.001** |
| **Exercise, n (%)** |  |  |  |  |  | 0.149 |
| Inactive | 0.18(9.17) | 0.04(8.27) | 0.05(7.59) | 0.06(10.79) | 0.03(10.76) |  |
| Active | 0.28(14.24) | 0.06(12.50) | 0.10(16.67) | 0.07(12.95) | 0.05(14.56) |  |
| Insufficient | 1.51(76.60) | 0.39(79.23) | 0.46(75.74) | 0.42(76.26) | 0.24(74.68) |  |
| **Medical History** |  |  |  |  |  |  |
| **Hypertension, n (%)** | 0.73(36.73) | 0.18(37.10) | 0.23(38.28) | 0.20(35.25) | 0.11(35.76) | 0.727 |
| **Dyslipidemia, n (%)** | 0.31(15.86) | 0.07(13.31) | 0.11(17.66) | 0.09(16.55) | 0.05(15.19) | 0.241 |
| **Diabetes, n (%)** | 0.33(16.62) | 0.07(14.31) | 0.10(16.50) | 0.10(17.63) | 0.06(18.67) | 0.351 |
| **Laboratory Data**^†^ |  |  |  |  |  |  |
| **ALT, U/L** | 21.06(16.91,29.14) | 20.47(15.97,27.47) | 21.64(17.17,29.83) | 20.35(16.83,28.17) | 23.38(18.09,31.80) | **<0.001** |
| **AST, U/L** | 19.59(16.48,23.59) | 19.65(16.25,23.75) | 20.04(16.90,24.14) | 19.29(16.42,23.30) | 19.27(16.02,22.43) | **0.016** |
| **Scr, umol/L** | 67.51(57.57,79.49) | 65.94(57.16,79.78) | 64.75(55.38,76.83) | 66.18(57.86,78.33) | 75.64(66.26,86.13) | **<0.001** |
| **BUN, mmol/L** | 5.61(4.66,6.65) | 5.58(4.61,6.59) | 5.62(4.68,6.58) | 5.64(4.74,6.81) | 5.60(4.63,6.57) | 0.571 |
| **TC, mmol/L** | 5.00(4.24,5.76) | 5.03(4.23,5.79) | 5.11(4.32,5.84) | 5.03(4.23,5.75) | 4.70(4.11,5.48) | **<0.001** |
| **TG, mmol/L** | 1.41(1.02,1.95) | 1.41(1.00,1.91) | 1.44(1.02,1.97) | 1.39(1.06,1.94) | 1.47(1.02,2.01) | 0.622 |
| **HDL-C, mmol/L** | 1.36(1.19,1.59) | 1.37(1.19,1.62) | 1.39(1.20,1.62) | 1.38(1.21,1.60) | 1.28(1.12,1.48) | **<0.001** |
| **LDL-C, mmol/L** | 2.92(2.42,3.39) | 2.98(2.45,3.44) | 2.96(2.48,3.42) | 2.89(2.38,3.42) | 2.81(2.32,3.31) | **0.018** |
| **FPG, mmol/L** | 5.68(5.29,6.41) | 5.63(5.25,6.28) | 5.71(5.31,6.51) | 5.67(5.26,6.42) | 5.81(5.33,6.55) | **0.026** |

Data were presented as median [p25, p75] or n [In thousand] (%). The Kruskal-Wallis test for continuous variables, and the chi-square test was employed for categorical variables to assess differences in the descriptive analyses. ^†^Presence of missing values. Bold text indicates statistical significance. ALT: Alanine aminotransferase; AST: Aspartate aminotransferase; Scr: Serum creatinine; BUN: Blood urea nitrogen; TC: Total cholesterol; TG: Triglyceride; HDL-C: High-density lipoprotein cholesterol; LDL-C: Low-density lipoprotein cholesterol; FPG: Fasting plasma glucose.

**Table S3** Univariate associations with triglyceride levels in the NHANES cohort.

| **Characteristic** | **Beta** | **95%CI** | ***P* value** |
| --- | --- | --- | --- |
| **Age, year** | 0.01 | -0.01, 0.01 | 0.066 |
| **Gender, n (%)** | -0.25 | -0.38, -0.12 | **<0.001** |
| **Race, n (%)** | -0.15 | -0.21, -0.09 | **<0.001** |
| **Education, n (%)** | -0.04 | -0.08, -0.01 | **0.013** |
| **family poverty income ration, %** | 0.02 | -0.01, 0.06 | 0.244 |
| **Smoke status, n (%)** | 0.03 | -0.04, 0.10 | 0.406 |
| **Alcohol status, n (%)** | 0.04 | -0.03, 0.11 | 0.245 |
| **Exercise, n (%)** | 0.01 | -0.08, 0.07 | 0.933 |
| **Depression, n (%)** | 0.17 | -0.06, 0.41 | 0.147 |
| **Hypertension, n (%)** | 0.26 | 0.15, 0.38 | **<0.001** |
| **Diabetes, n (%)** | 0.36 | 0.18, 0.53 | **<0.001** |

Bold text indicates statistical significance.

**Table S4** Univariate associations with triglyceride levels in the Urumqi cohort.

| **Characteristic** | **Beta** | **95%CI** | ***P* value** |
| --- | --- | --- | --- |
| **Age, year** | -0.02 | -0.02, -0.01 | **<0.001** |
| **Gender, n (%)** | -0.10 | -0.22, 0.03 | 0.126 |
| **Education, n (%)** | 0.03 | -0.04, 0.10 | 0.433 |
| **family income, thousand** | 0.01 | 0.00, 0.01 | 0.933 |
| **Smoke status, n (%)** | 0.06 | -0.04, 0.15 | 0.269 |
| **Alcohol status, n (%)** | 0.26 | 0.10, 0.43 | **0.002** |
| **Exercise, n (%)** | 0.01 | -0.10, 0.01 | 0.964 |
| **Hypertension, n (%)** | 0.01 | -0.12, 0.13 | 0.972 |
| **Diabetes, n (%)** | 0.10 | -0.06, 0.26 | 0.230 |

Bold text indicates statistical significance.

**Table S5** The association between unprocessed beef intake and triglyceride levels in the NHANES cohort.

| **NHANES** | **Beta(95%CI), *P* value** |  |  |
| --- | --- | --- | --- |
|  | **Model 1** | **Model 2** | **Model 3** |
| **Beef Intake(continuous)** | 0.12 (0.03, 0.20), **0.008** | 0.10 (0.02, 0.18), **0.014** | 0.11 (0.03, 0.20), **0.012** |
| **Beef Intake (quartiles)** |  |  |  |
| **Quartile 1** | Reference | Reference | Reference |
| **Quartile 2** | 0.03 (-0.11, 0.16), 0.704 | 0.01 (-0.13, 0.14), 0.947 | -0.05 (-0.19, 0.09), 0.506 |
| **Quartile 3** | 0.19 (0.04, 0.35), **0.016** | 0.17 (0.02, 0.32), **0.026** | 0.17 (0.00, 0.34), 0.055 |
| **Quartile 4** | 0.28 (0.09, 0.47), **0.004** | 0.26 (0.08, 0.43), **0.005** | 0.26 (0.08, 0.43), **0.005** |

Bold text indicates statistical significance.

Model 1: No covariates were adjusted.

Model 2: Age, gender, race were adjusted.

Model 3: Age, gender, race, education level, PIR, smoking status, alcohol status, exercise status, medical history of depression, hypertension, diabetes were adjusted.

95%CI: 95% Confidence Interval.

**Table S6** The association between beef intake and triglyceride levels in the Urumqi cohort

| **Urumqi** | **Beta(95%CI), *P* value** |  |  |
| --- | --- | --- | --- |
|  | **Model 1** | **Model 2** | **Model 3** |
| **Beef Intake(continuous)** | 0.16 (0.05, 0.27), **0.006** | 0.13 (0.01, 0.24), **0.027** | 0.12 (0.01, 0.23), **0.046** |
|  |  |  |  |
| **Beef Intake (quartiles)** |  |  |  |
| **Quartile 1** | Reference | Reference | Reference |
| **Quartile 2** | 0.07 (-0.10, 0.23), 0.431 | 0.07 (-0.10, 0.23), 0.432 | 0.06 (-0.10, 0.22), 0.485 |
| **Quartile 3** | 0.04 (-0.13, 0.21), 0.643 | 0.02 (-0.15, 0.18), 0.823 | 0.01 (-0.15, 0.18), 0.877 |
| **Quartile 4** | 0.27 (0.08, 0.47), **0.005** | 0.23 (0.02, 0.43), **0.029** | 0.20 (-0.01, 0.40), 0.057 |

Bold text indicates statistical significance.

Model 1: No covariates were adjusted.

Model 2: Age, gender were adjusted.

Model 3: Age, gender, education level, family income, smoking status, alcohol status, exercise status, medical history of hypertension, diabetes were adjusted.

95%CI: 95% Confidence Interval.

**Table S7** Mendelian randomization analysis of beef intake and triglyceride levels.

| **Outcome** | **MR method** | **Beta** | **SE** | **95%CI^*^** | ***P* value** | **Heterogeneity** | | **Pleiotropy** | | ***P* value for MR-PRESSO global test** |
| --- | --- | --- | --- | --- | --- | --- | --- | --- | --- | --- |
|  |  |  |  |  |  | **Q statistics** | ***P* value** | **Intercept** | ***P* value** |  |
| ebi-a-GCST90092992 | IVW | 0.769 | 0.195 | 0.387, 1.151 | 0.001 | 48.907 | 0.671 | 0.004 | 0.124 | 0.701 |
|  | MR-Egger | -0.361 | 0.749 | -1.829, 1.107 | 0.632 |  |  |  |  |  |
|  | Weighted median | 0.613 | 0.283 | 0.058, 1.168 | 0.031 |  |  |  |  |  |
|  | Weighted mode | 0.868 | 0.608 | -0.324, 2.060 | 0.159 |  |  |  |  |  |
| met-d-Total_TG | IVW | 0.743 | 0.195 | 0.361, 1.125 | 0.001 | 51.541 | 0.569 | 0.004 | 0.111 | 0.586 |
|  | MR-Egger | -0.433 | 0.752 | -1.907, 1.041 | 0.567 |  |  |  |  |  |
|  | Weighted median | 0.616 | 0.288 | 0.052, 1.180 | 0.001 |  |  |  |  |  |
|  | Weighted mode | 0.733 | 0.587 | -0.417, 1.883 | 0.218 |  |  |  |  |  |

^*^The 95%CI for the effect estimate is calculated based on the standard error using the normal approximation method (i.e., beta±1.96*se), which is derived from Neyman's confidence interval theory (Neyman, 1937).
